# Valedictory Address

**Published:** 1867-07

**Authors:** R. E. McVey

**Affiliations:** President of the Morgan County Medical Society


### ARTICLE XXXI.

### VALEDICTORY ADDRESS.

By R. E. McVEY, M.D., President of the Morgan County Medical Society.

Delivered at the Anniversary Meeting, May 9th, 1867.

Gentlemen:—One year ago a few of us met in the court house in this place for the purpose of organizing a medical society, that the physicians of Morgan County might become workers in that great medical organization of our country which extends from the shores of the Atlantic to the Pacific, and from the great lakes to the Gulf of Mexico.

What is the object of this vast organization, with its thousands of auxilliaries? It is a philanthropic one, to minister to mankind in affliction, and that we may the better be enabled to do this, to collate and disseminate such information as each may acquire in his practice throughout the profession—to open new leads in the mines of science and extract unrevealed virtues from the ores already dug.

In this organization the man of limited attainments is permitted to share the ripe fruit gathered by the scientific research and experience of those of eminent culture, and I am proud to know that in this association we can point to some bright, particular stars, whose lights wane not by the side of any in the medical firmament. Great, good, and learned men, whose influence is not confined by professional boundaries, but permeates society at large; and I hope it will not be regarded as an invidious allusion if I here mention the name of our own David, who has wrestled so successfully with the Goliahs of deformities.

It is by the contact of mind with mind that our intellects are burnished, and the privilege we here enjoy of discussing the various medical questions of the day, may be instrumental in developing new theories, in enlarging the area of thought, and fit us to grapple with error in its many forms; and from the garners of the medical harvest aid us in separating the chaff from the wheat, that we may accept only that which is good, rejecting all that is bad. Here every man comes bringing his sheaves—some full of the brambles of dogmatism, others bristling with the thistles of crude and ill-digested theories, but still more heavy only with the golden grain of ripe experience. It is from these organizations that the materials are gathered for our medical journals. They are the carriers of our thoughts to the people, and should be found in the hands of every respectable practioner of medicine. The influence of our associations would be circumscribed indeed, were it not for those brethren of that profession, who hold in their hands tne lightnings of the press, that mighty engine of civilization without which science to-day would still wear the monk's garb of the dark ages, and be as powerless for good as Prometheus bound.

Until the ingenious inventor of types opened the highway of thought throughout the world—mind chafed as a fretted prisoner within cloister walls, and was manacled with the rusty chains of uncertain traditions—but now mounted upon a chariot as glorious as that of Ezekiel's vision, with the lightning as a servant and out-rider, it is moving grandly to the accomplishment of man's high destiny. To our profession and through us to the whole human family, this man of the press has come with healing in his wings, and it is but fitting that on all proper occasions we should acknowledge the obligation.

Gentlemen, our profession has not yet reached the zenith of its glory. That is a goal situated on the outer boundaries of that promontory where human reason exhausted, shall lie down to slake its thirst in springs which have their source in another world than ours. But day by day, the great work advances, and by the patient toil and combined efforts of the profession, we may, at least approximate that perfection which it is not given humanity to attain.

To the studious and energetic, inquiry will continue to yield her trophies until the science of life shall be reduced to simple laws. But the labor before us is well calculated to discourage the most hopeful and enthusiastic practioner of medicine.

We have not only the natural weaknesses and tendency of the body to decay to combat, for these we might provide with an accuracy and success almost equal to that with which the builder props and strengthens the crumbling edifice—but we have to contend with flames of consuming appetites, undermining habits of luxury, and the thousand and one forms of vice with which the human frame is ever being assailed, and which have their origin in moral infirmities, which it is not our province to correct.

Only at the millenium will the complications of our profession be resolved into plain reasoning from purely natural causes to their effects.

But if our work is endless, our record is nevertheless sure, and by every dictate of human sympathy we are impelled to increased exertion in behalf our fellow-man.

Let us neglect, then, none of the means within our reach for the advancement of the profession which we have so nearly at heart, and the honor and fame of which we have voluntarily taken upon ourselves to sustain.

Our responsibility is very great, and I should regard that physician recreant, indeed to the important trust, who should fail to contribute to the maintenance, usefulness and interests of these county associations.

We are all satisfied that the short life of our society has been productive of much good, yet how much more prolific of beneficient results may it be made in the future. Will each member here to-day, commemorating its first annivesary, pledge himself to aid in extending its influence, that our next annual meeting may exhibit signs of prosperity even more marked than at present.

In recalling the meetings of the past, I remember with pleasure the spirit of harmony which ever prevailed, and doubt not the discussions have been quite as profitable to all as to myself; and I here take occasion to express my sense of gratitude to all who have participated in those meetings as essayists and speakers for suggestions which will not soon be forgotten.

In vacating the chair I feel that I am making room for a more able occupant, one who will guide your deliberations with a more skillful hand. I have endeavored to discharge my duties faithfully, but I now feel that my experience in parliamentary rules but illy fitted me for the position. Your kindness in elevating me to the presidency, however, has been greatly exceeded by your manly forbearance with all my mistakes. Is it necessary for me to thank you now for this courtesy? I have mentally done so a thousand times while sitting as your presiding officer.

I shall never cease to prize the honor which your votes have given me—that of being your first president.

---

To Graduates of Chicago Medical College.—Those graduates of Chicago Medical College who have not received a copy of the Constitution and By-Laws of the Alumni Association, recently formed, will please send in their address to the Sec'y,

S. A. McWILLIAMS,

*166 State Street, Chicago.*

